# Challenges Faced by Highly Polyploid Bacteria with Limits on DNA Inheritance

**DOI:** 10.1093/gbe/evab037

**Published:** 2021-03-02

**Authors:** Esther R Angert

**Affiliations:** Department of Microbiology, Cornell University, New York

**Keywords:** *Epulopiscium*, endospore, intracellular offspring, comparative genomics, fish gut microbiota

## Abstract

Most studies of bacterial reproduction have centered on organisms that undergo binary fission. In these models, complete chromosome copies are segregated with great fidelity into two equivalent offspring cells. All genetic material is passed on to offspring, including new mutations and horizontally acquired sequences. However, some bacterial lineages employ diverse reproductive patterns that require management and segregation of more than two chromosome copies. *Epulopiscium* spp., and their close relatives within the Firmicutes phylum, are intestinal symbionts of surgeonfish (family Acanthuridae). Each of these giant (up to 0.6 mm long), cigar-shaped bacteria contains tens of thousands of chromosome copies. *Epulopiscium* spp. do not use binary fission but instead produce multiple intracellular offspring. Only ∼1% of the genetic material in an *Epulopiscium* sp. type B mother cell is directly inherited by its offspring cells. And yet, even in late stages of offspring development, mother-cell chromosome copies continue to replicate. Consequently, chromosomes take on a somatic or germline role. *Epulopiscium* sp. type B is a strict anaerobe and while it is an obligate symbiont, its host has a facultative association with this intestinal microorganism. Therefore, *Epulopiscium* sp. type B populations face several bottlenecks that could endanger their diversity and resilience. Multilocus sequence analyses revealed that recombination is important to diversification in populations of *Epulopiscium* sp. type B. By employing mechanisms common to others in the Firmicutes, the coordinated timing of mother-cell lysis, offspring development and congression may facilitate the substantial recombination observed in *Epulopiscium* sp. type B populations.

**Significance**

The giant bacterium *Epulopiscium* sp. type B maintains tens of thousands of copies of its genome throughout its lifecycle. These highly polyploid bacteria reproduce solely by the formation of internal offspring. A limited number of genome copies is passed on to each offspring, but most copies remain in the mother cell and are needed to support metabolism of the mother cell and growing offspring. Since not all copies of the genome are inherited, *Epulopiscium* populations run the risk of losing genetic diversity acquired in the somatic genome copies. *Epulopiscium* sp. type B populations bypass this loss by the coordinated release of DNA from dying mother cells and recombination of these fragments in genome copies within newly emerged offspring cells.

## Populations of Bacteria That Exhibit a Germline/Soma Division

Most scientists do not think about bacteria when considering germline dynamics. That is entirely reasonable because most of our models for bacteriology use binary fission for reproduction. In binary fission, a bacterium grows to about twice its starting size, while it replicates and then segregates copies of its genome to distal parts of the cell. Reproduction culminates in division of the cytoplasm at the midcell to produce two equivalent and self-sufficient offspring cells, each with a complete complement of the parent cell’s genomic material. All genetic material is preserved in this process.

Several, perhaps less-familiar bacterial models have communication systems used to coordinate multicellular behaviors and regulate alternative reproductive programs (Angert 2005; Claessen et al. 2014). A number have independently evolved developmental processes that allow cells to specialize, with some members of a population transforming into highly dispersible propagules. In these patterns of development, the propagules (usually spores) could be considered the “germline” of a *population* or *colony* of cells.

Members of the myxobacteria (phylum Proteobacteria), such as *Myxococcus xanthus*, exhibit multicellular behaviors for finding prey and for dispersing myxospores when nutrients become limited (Bretl and Kirby 2016; Keane and Berleman 2016). Myxobacteria are motile, typical-looking, rod-shaped bacteria that live in the soil or in detritus. Cells can live as independent operators, seeking out and finding prey bacteria to lyse and consume. Members of a population of *M. xanthus* can alternatively coordinate their activities and even exhibit a strategy in which the population organizes into a “bacterial wolfpack” to hunt down, kill and consume large numbers of prey bacteria (Berleman and Kirby 2009). When prey or nutrients are depleted, cells send out additional peptide and amino acid-based signals to help redirect their behavior. These signals may assist with a quorum sensing checkpoint to ensure enough cells are in the vicinity to engage in the development of a fruiting body (Kroos 2017). The *M. xanthus* population aggregates and cells within the aggregate begin to differentiate. The majority of cells in the responding population become the structural and/or nutritional support used to erect the fruiting body, which rises above the surface of the soil. Housed within, a subset of cells (∼10% of the aggregate) differentiates into myxospores (Kroos 2017). The structural cells of the fruiting body eventually die, while the resilient spores are carried off to start over in a new site.

In contrast, the filamentous growth of *Streptomyces coelicolor*, and related genera in the phylum Actinobacteria, keeps cells of the colony physically connected to one another throughout vegetative growth and arthrospore development (Barka et al. 2016; Chater 2016). *Streptomyces* spp. are common free-living, saprophytic soil microbes, although many species in this group form symbiotic relationships with eukaryotic organisms (Lewin et al. 2016). Streptomycetes are known for their ability to produce diverse secondary compounds, including bioactive compounds we use as antibiotics (Barka et al. 2016). At the start of vegetative growth, the single-nucleoid containing spore germinates. A germ tube emerges and the vegetative hypha extends into its surroundings by tip growth (Chater 2016). DNA replication within the hyphal filament and infrequent division of the cytoplasm leads to the formation of long, multi-nucleoid cells (Flardh and Buttner 2009). The complex, branched network of hyphae that comprises the substrate mycelium spreads out to find and extract nutrients from the local environment. The success of the sessile colony relies on its keen ability to sense environmental change and respond in an appropriate manner (McCormick and Flardh 2012). As nutrients are depleted, a developmental shift begins. Responses of individual cells within the colony are dictated not only by their position within the colony but also by the production and reception of peptide signals that coordinate the colony response (McCormick and Flardh 2012). Hyphae on the surface of the colony become enrobed in sheaths composed of hydrophobic peptides which allows these unbranched filaments to break any surface tension and rise above the substrate producing a forest of aerial hyphae (Flardh and Buttner 2009; McCormick and Flardh 2012). The terminal cell of an aerial hypha differentiates. Tip growth stops and rounds of replication may lead to the production of 50 or more copies of the genome within the terminal cell (Flardh and Buttner 2009). The nucleoids condense, and the terminal cell undergoes simultaneous, multiple division to produce a chain of small, single-nucleoid cells. These cells develop into spores. Meanwhile, the colony may produce a variety of secondary compounds, as the substrate mycelial cells undergo a form of programmed cell death (Barka et al. 2016). The antimicrobials may reserve nutrients in the lysate for the colony to support spore development by preventing consumption of these resources by motile competitors. The substrate mycelium is sacrificed for the production of highly dispersible propagules.

The processes by which cells are “chosen” to become spores in a population or colony are not known, but it appears that the process is not random for Streptomycetes. The production of spores only in terminal cells of aerial hyphae could diminish the genetic diversity of the next generation. The exceptionally efficient conjugal transfer of DNA and high frequency of genetic exchange observed in Streptomycetes (Chater 2016) may allow these populations to compensate for this potential loss in diversity.

**Fig. 1:**
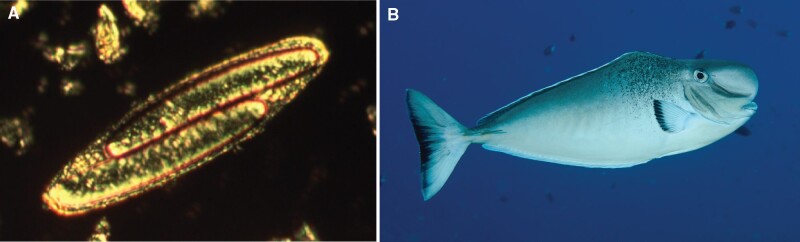
*Epulopiscium* sp. type B is an obligate symbiont of the surgeonfish *Naso tonganus*. (A) *Epulopiscium* sp. type B, also known as “*Ca.* Epulopiscium viviparus.” The two large structures within this *Epulopiscium* are offspring. (B) The surgeonfish *Naso tonganus* inhabits tropical coral reef systems and its native range includes the Great Barrier Reef. *Epulopiscium* sp. type B is often, but not always, found in high numbers in the intestinal tract of *N. tonganus*. The photo of *N. tonganus* was taken by Kendall Clements.

The focus of this review is bacteria in the Firmicutes phylum that establish a germline/soma division within an individual cell as part of its normal life cycle. These bacterial giants (fig. 1) are called *Epulopiscium* spp. and their relatives, also known as “epulos” (Clements et al. 1989). To date, no member of this group of intestinal symbionts of surgeonfish (family Acanthuridae) has been cultured in the laboratory and what we know about the biology of these microorganisms comes from observations and analyses of cells collected from wild surgeonfish. In these large and extremely polyploid bacteria, only some genome copies are inherited by offspring. The majority of the genome copies are not directly passed on as intact chromosomes. In some ways, this pattern of genomic inheritance resembles the colony and population level responses described above, except in *Epulopiscium* spp. the germline/soma divide happens within a single cell. Like Streptomycetes, the selection of the germline in *Epulopiscium* spp. may compromise diversity of future generations.

## Evolution of a Novel Form of Bacterial Reproduction in *Epulopiscium* spp.

The largest *Epulopiscium* cells are cigar-shaped and can reach lengths in excess of 0.6 mm (Hutchison et al. 2014). When they were first discovered, the size of *Epulopiscium* cells, their complex ultrastructure and the manner in which they reproduce led to their taxonomic placement in the Eukarya domain, though their affiliation with any known protist was not apparent (Fishelson et al. 1985). The symbionts were located in the intestinal tract of several species of surgeonfish collected from coral reef ecosystems in the Red Sea. These putative protists were given the name *Epulopiscium fishelsoni* (Montgomery and Pollak 1988). Their specific association with herbivorous species of surgeonfish led to the hypothesis that these giant microbes play a role in host nutrition (Fishelson et al. 1985). Later, surveys of the gut microbiota of marine fish collected in the south Pacific revealed a great variety of similar intestinal symbionts, and further supported the link between these intestinal microbes and primarily herbivorous or detritivorous surgeonfish species (Clements et al. 1989). Phylogenetic studies based on small subunit ribosomal RNA gene sequences demonstrated that epulos are Bacteria, and that *E. fishelsoni* is a complex of at least two distinct genera (Angert et al. 1993). Epulos form a clade within the Firmicutes phylum, Clostridia class, and the Lachnospiraceae family (Flint et al. 2005; Miyake et al. 2016). The epulo group comprises diverse bacteria from small, micron-long rods to giant cigar-shaped cells.

**Fig. 2:**
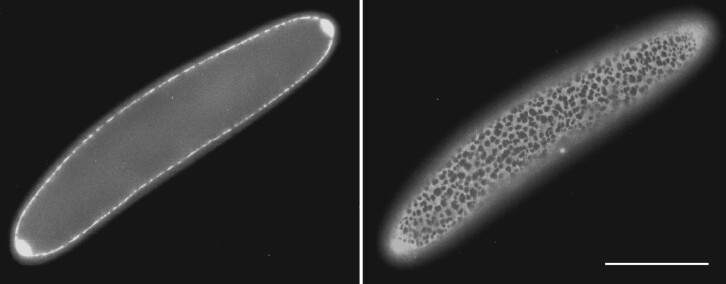
DNA in *Epulopiscium* sp. type B forms a network beneath the cytoplasmic membrane. These images show two different focal planes of the same DAPI-stained cell. The image on the left is a medial focal plane. To the right is a focal plane just under the cell surface. In large cells, most of the DNA is located in a layer at the cytoplasmic periphery, just under the cell membrane. The two bright structures at the poles of this cell are offspring. Note that only a portion of the mother-cell DNA is directly inherited. Scale bar represents 50 µm. Reprinted with permission from (Robinow and Angert 1998).

The unusual cell and reproductive biology of the largest *Epulopiscium* spp. contributed to some of the early confusion about the fundamental nature of these cells. When viewed with an epifluorescence microscope, cells stained with the DNA-specific dye 4′,6-diamidino-2-phenylindole (DAPI) appeared to contain one or two well-defined nuclei (Montgomery and Pollak 1988). These ostensible nuclei were inconstant and a nuclear envelop was never observed in thin sections of cells viewed with transmission electron microscopy. Differences in the sizes of nuclei and the observed location of DNA in cells were interpreted as changes leading to the development of offspring around segregated nuclei. Closer inspection revealed the diffuse staining of cellular DNA in a fine network just under the cytoplasmic membrane, entirely encasing the cytoplasm (fig. 2). The intense nucleus-like staining pattern described in early studies came from the tightly packed DNA within small, intracellular offspring cells (Robinow and Kellenberger 1994; Robinow and Angert 1998).

A remarkable feature of giant *Epulopiscium* cells is that intracellular offspring development follows a daily cycle (Montgomery and Pollak 1988). *Epulopiscium* sp. type B (“*Ca.* Epulopiscium viviparus”), a symbiont of the surgeonfish *Naso tonganus* (fig. 1), is the most studied *Epulopiscium* species (Angert et al. 1993; Angert and Clements 2004; Mendell et al. 2008; Ward et al. 2009; Miller et al. 2011, 2012; Hutchison et al. 2018; Arroyo et al. 2019). These phylogenetically distinct and often abundant populations of intestinal symbionts have been used as a model for studying the cellular and developmental biology of giant bacteria.

*Epulopiscium* sp. type B cells are usually 100–300 µm long and they reproduce once every day. Timing of offspring growth parallels the diurnal feeding pattern of their host. As a result, a population of cells collected from single fish is well synchronized with respect to offspring development (Angert and Clements 2004; Mendell et al. 2008; Miller et al. 2011). How developmental progression is coordinated within a population is currently unknown.

Compared with other members of the Firmicutes, an extraordinary proportion of the *Epulopiscium* sp. type B genome is dedicated to carbohydrate transport and metabolism (Miller et al. 2012). This supports the hypothesis that these gut symbionts provide nutritional benefits to their host by degrading recalcitrant polysaccharides in the algae that the fish consumes. The simple sugars and sugar alcohols released are fermented by *Epulopiscium* and other gut microbes, which produces short chain fatty acids, such as acetate, that can be used by the host (Clements and Choat 1995; Clements 1997). Furthermore, the daily and coordinated lysis of mother cells can release organic nutrients (nucleic acids, proteins, vitamins, etc.) into the intestinal lumen, which could provide a benefit to other microbial residents and the host but in a manner that does not compromise the *Epulopiscium* population (Ward et al. 2009).

**Fig. 3:**
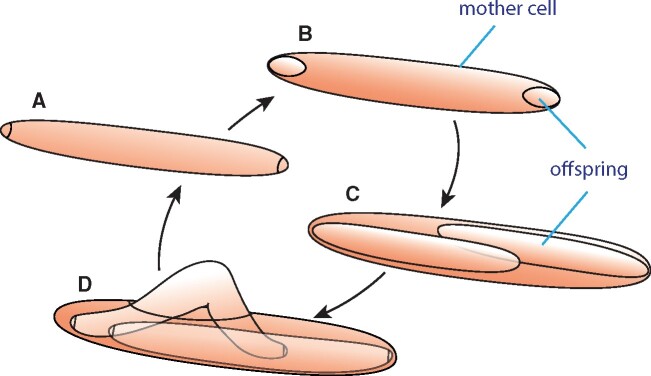
The *Epulopiscium* spp. life cycle produces internal offspring once every 24 h. (A) The two polar offspring primordia are engulfed by the mother cell. (B) The polar offspring are contained within a membrane-bound compartment in the cytoplasm of the mother cell. (C) Offspring cells grow within the mother cell until they nearly fill the mother cell. (D) The offspring are released through a tear in the mother-cell envelop. The mother cell is killed in this process. In *Epulopiscium* sp. type B, asymmetric division usually occurs in offspring (to produce “granddaughter cells”) prior to their release from the mother cell.

One of the first visual changes in an *Epulopiscium* cell as reproduction begins is the coalescence of DNA at both poles of the cell (Angert and Clements 2004). Compared to most of the mother-cell DNA, the polar DNA is highly condensed and often appears tethered to the inner cell poles (Robinow and Angert 1998; Hutchison et al. 2018). Generally, *Epulopiscium* sp. type B divides near both cell poles (fig. 3), trapping some of the pole-associated DNA within each tiny polar offspring primordium (Angert and Clements 2004). It has been estimated that only about 1% of the total DNA in a mother cell is packaged into offspring cells. The polar primordial cells are engulfed by the mother cell. Offspring grow within a membrane-bound compartment in the cytoplasm of the mother cell. It takes only a few hours of growth for the intracellular offspring to nearly fill the mother-cell cytoplasm. Late in the offspring growth cycle, the mother cell appears to undergo a programmed cell death (Ward et al. 2009). DNA in the mother cell diminishes, and the mother-cell envelope splits open to release its offspring. All that appears to be left behind after offspring release is an empty shell (Montgomery and Pollak 1988).

The offspring developmental program in *Epulopiscium* spp. shares morphological features with endospore formation in other members of the Firmicutes, including extreme asymmetric division and engulfment of a polar cell (Angert et al. 1996). Gene-based analyses have shown that this unusual reproductive program is a derived form of endosporulation (Flint et al. 2005; Miller et al. 2011, 2012). However, in *Epulopiscium* sp. type B, the ability to produce mature, phase-bright endospores has been lost. Endospore formation is a complex developmental program that requires the coordinated progression of divergent genetic cascades in the developing spore (forespore) and within its mother cell (Khanna et al. 2020). In *Bacillus subtilis* and spore-forming *Clostridium* spp., this requires modulation of the expression of hundreds of genes (Miller et al. 2012; Al-Hinai et al. 2015). The core sporulation program, conserved among all endospore-forming members of the Firmicutes, is comprised of approximately 60 genes (Galperin et al. 2012), and another 80 genes are widely conserved (Miller et al. 2012). Collectively the sporulation programs of all spore forming Firmicutes (the sporulation pangenome) is enormous but poorly characterized. Comparative studies of endospore developmental programs have shown that this complex genetic program has been fodder for selection that has tuned the process to the needs of specific lineages (Galperin et al. 2012; Miller et al. 2012; Hutchison et al. 2014; Al-Hinai et al. 2015; Davidson et al. 2018). In some, it has transformed from a standby survival program to an effective reproductive process.

Genome comparisons have shown that *Epulopiscium* sp. type B has almost all of the highly conserved genes needed for endospore formation in spore-forming Clostridia and Bacilli (Miller et al. 2012). For example, the *Epulopiscium* sp. type B genome codes for the master regulator Spo0A, a linchpin transcription factor that when activated starts the sporulation program (Miller et al. 2012; Yutin and Galperin 2013; Davidson et al. 2018). *Epulopiscium* sp. type B contains other key transcription factors and all sporulation-specific sigma factors. The engulfment machinery is also conserved. However, some genes required for spore maturation appear to be missing. *Cellulosyliticum lentocellum* is one of the closest known endospore-forming, cultured relatives of the *Epulopiscium* group (Miller et al. 2012; Hutchison et al. 2018). When the genome content of *C. lentocellum* and *Epulopiscium* sp. type B were compared, only one of the nine genes that *C. lentocellum* has for the synthesis and transport of dipicolinic acid (DPA) could be located in the *Epulopiscium* sp. type B genome (Miller et al. 2012). DPA could be considered a signature molecule of endospores. DPA is synthesized in the mother cell and transported into the developing spore where it forms a calcium–DPA complex (Setlow et al. 2006). The accumulation of calcium–DPA and concurrent reduction in water within the cytoplasm are responsible for the phase-bright appearance of mature endospores and their resistance to wet heat. The phase-bright spores of endospore producing epulos contain large amounts of DPA as well (Flint et al. 2005). Arguably, endospore formation is a survival strategy that enhances resistance to environmental assaults and improves the dispersal of many members of the Firmicutes that are strict anaerobic gut symbionts (Klaasen et al. 1992; Angert and Losick 1998; Browne et al. 2016; Moeller et al. 2016). For now, it is uncertain why *Epulopiscium* sp. type B lost its ability to make mature spores while other epulos have not (Flint et al. 2005).

The model for studying the genetics and biochemistry of endosporulation is the soil bacterium *B. subtilis* (Bate et al. 2014; Khanna et al. 2020). The majority of endospore-forming Bacilli normally produce a single endospore when a cell is nutrient stressed (Setlow and Johnson 2019; Khanna et al. 2020). The program begins only when options for finding alternative resources have been exhausted (Lopez and Kolter 2010). During normal growth, *B. subtilis* uses binary fission for reproduction. As a starving *B. subtilis* cell enters sporulation, it suppresses chromosome replication initiation (Setlow and Johnson 2019). Assembly of the cell division complex at the midcell is blocked, and instead, division complexes form at both cell poles. Only one of these polar complexes becomes fully active, leading to asymmetric division (Khanna et al. 2020). It has been suggested that the other division complex may act as a failsafe if the first attempt at asymmetric division fails. Two copies of the chromosome are present in the *B. subtilis* sporangium as the cell divides asymmetrically to form the mother cell and smaller forespore. One chromosome copy is packaged into the tiny forespore while the other remains in the mother cell. Both are necessary for expression of the divergent genetic programs that play out in each cell type as spore development progresses.

Mutations in certain early sporulation genes can cause division at both cell poles (Eichenberger et al. 2001). In these mutants, the two chromosome copies are packaged into the two polar forespores, leaving the mother cell devoid of a genome. Without the genetic contributions of the mother cell, sporulation arrests prior to forespore engulfment. Surprisingly, only two genetic changes in the *B. subtilis* are needed to convert it to a viable disporic (Eldar et al. 2009). One relaxes the repression of DNA replication initiation during sporulation so that three or more chromosome copies are present in the sporangium. The other change relaxes controls over the cell division machinery to allow bipolar division.

These genetic modifications, and likely others, come into play in wildtype, multiple endospore-forming bacteria such as *Metabacterium polyspora*. In this bacterium, binary fission appears to be a vestigial form of reproduction, which largely has been replaced by formation of multiple endospores (Angert and Losick 1998). This shift toward reliance on sporulation may help reinforce the symbiotic association of this gastrointestinal symbiont with a guinea pig by taking advantage of the coprophagous behavior of its host. Despite being anaerobes, the mature and metabolically quiescent spores of *M. polyspora* are not affected by oxygen encountered outside of the guinea pig intestinal tract. Thus, reproduction by sporulation prepares the symbiont for cycling out of and back into the host intestinal tract. As the host consumes its feces, the *M. polyspora* spores survive passage through the mouth and stomach, and then germinate in the small intestine. Often, germinating spores bypass binary fission and immediately begin the next cycle of sporulation. The *M. polyspora* mother cell divides at both poles producing two viable forespores (Angert and Losick 1998). Genomic DNA continues to replicate during sporulation in the mother cell and within developing forespores (Ward and Angert 2008). To form more than two endospores, fully engulfed forespores may undergo a form of binary fission (Angert and Losick 1998).

*Epulopiscium* sp. type B appears to have further modified the highly conserved endosporulation program into a novel, and required, mode of cellular reproduction (Angert et al. 1996). Remarkably, evidence of binary fission has never been observed in *Epulopiscium* sp. type B. Phase-bright offspring that resemble mature endospores have also never been detected. Based on genome content, *Epulopiscium* sp. type B appears incapable of producing mature endospores (Miller et al. 2012), and likely their offspring are much less resilient than endospores. Instead of cycling in and out of the host frequently, like *M. polyspora*, *Epulopiscium* sp. type B populations reside in the host fish over many reproductive cycles which may have relaxed selection to maintain the ability to produce mature spores (Angert and Losick 1998). Like *M. polyspora*, an *Epulopiscium* sp. type B cell divides at both poles (Angert and Clements 2004). Only a small proportion of the mother cell genomic resources are partitioned into the newly formed offspring primordia. *Epulopiscium* sp. type B often produces two offspring but it can produce as many as 12 (Hutchison et al. 2014). How more than two intracellular offspring are formed is still mysterious. The regulators or conditions that support different numbers of offspring are unknown. It is also not known if *Epulopiscium* sp. type B might undergo binary fission under certain conditions since they do assemble a fully functional division complex at each cell pole (Angert and Clements 2004).

## Evidence of Extreme Polyploidy in Giant Bacterial Cells

Bacterial and archaeal model organisms are generally small and highly efficient organisms that grow well in laboratory culture. These small cells have a high surface area relative to their cytoplasmic volume to maximize diffusive exchange of molecules with their environment and timely movement of biomolecules within the cytoplasm (Koch 1996; Schulz and Jorgensen 2001; Westfall and Levin 2017). Cell size is tightly regulated; it is essential for a cell to maintain enough volume to contain everything it needs for survival, but it must remain small enough to keep the relative surface area high. Space is at a premium. Consequently, bacterial and archaeal cells are often under selection to maintain one or only a few copies of their genomes. Alternatively, selection for specific metabolic functions or cell attributes may lead to the amplification of parts of a chromosome (van der Woude and Baumler 2004; Blount et al. 2020).

It has come to light in recent years that many bacteria and archaea, will maintain at least two genome copies at all times while others maintain multiple copies or are polyploid (Angert 2012). Maintaining multiple genome copies is valuable and may serve a variety of functions for bacteria and archaea (Zerulla and Soppa 2014). Additional chromosome copies may provide intact templates for DNA repair in stressful environments that are inherently damaging due to ionizing radiation or oxidative stress (Ohbayashi et al. 2019). Polyploidy is seen in obligate and facultative intracellular symbionts (Moran et al. 2005; Mergaert et al. 2006). The additional genome copies may help support the metabolic demands of the eukaryotic host. It has been suggested that copies of the genome may serve as an organic phosphate reserve, and extra chromosome copies may be dismantled and used as needed (Soppa 2014). Polyploidy in bacteria can impact the timing that newly acquired mutations are fully expressed as newly acquired mutations proliferate in the cell (Sun et al. 2018).

Eukaryotic examples of polyploidy or gene amplification are widespread and vary in their function. Polyploidy is important to support elevated expression needs and is seen in bacteriocytes (that host intracellular symbionts), in specialized cells (from hepatocytes to insect salivary glands) and in cancer cells (supporting aberrant growth and seen in cells responsible for recurrence), just to name a few examples (Shu et al. 2018; Donne et al. 2020; White-Gilbertson and Voelkel-Johnson 2020). Polyploidy may be a response to stress, as it expands the genomic options for adaptation (Fox et al. 2020). Whole genome duplication has been an important contributor to the evolution of fungi, animals and plants (Otto and Whitton 2000; Priest et al. 2020). One of the most complex genomic resource management systems known has been described in Ciliates (Prescott 2000). Here the micronucleus (germline) contains a single copy of the genome. The cell guards this copy in a transcriptionally inactive state. The macronucleus is transcriptionally active and contains an amplified subset of genes (Pilling et al. 2017). Examples of polyploidy or regional genetic amplification are known across the tree of life and these genomic configurations can provide immediate cellular benefits as well as offer substantial evolutionary options.

*Epulopiscium* sp. type B cells contain an astonishing amount of DNA. When examined under high magnifications, the stained DNA appeared to be the equivalent of thousands of bacterial nucleoids interconnected in a web-like structure (Robinow and Angert 1998). Quantifying DNA extracted from collections of cells revealed that large mother cells with large internal offspring contained on average 250 pg of DNA, while smaller mother cells with tiny offspring contained an average of 85 pg (Mendell et al. 2008). In comparison, the genome of *Escherichia coli* K-12 is approximately 4.6 fg and a human diploid cell would contain approximately 6 pg of DNA. The exceptional amount of DNA in *Epulopiscium* sp. type B begged the questions: why does a bacterium need so much DNA and what is the makeup of this cellular DNA (Mendell et al. 2008)? This particular giant bacterium is packed full of ribosomes, it has a high metabolic rate and offspring growth happens rapidly during a defined period of the day. The massive amount of cellular DNA is likely needed for the timely expression of genes to support the metabolic demands of rapid growth and offspring development. The DNA may also reinforce the host/symbiont relationship by allowing for a more substantial contribution to the host. It has been hypothesized that the predictable and abundant carbon and energy sources supplied to *Epulopiscium* cells by their host fish may be key to supporting these genomic resources (Angert 2012). Addressing the question about the composition of the DNA was challenging, especially in a bacterium that could not be grown in pure culture. If *Epulopiscium* sp. type B had a genome that was similar in size to most other bacterial genomes (1–10 Mbp), large *Epulopiscium* cells could contain thousands of genome copies (Mendell et al. 2008). However, it was also possible that the genome structure of *Epulopiscium* sp. type B had been modified to better support specific metabolic needs. Portions of the genome that contain genes essential for rapid growth could be amplified. For many microorganisms, the genome size of an isolate can be estimated from banding patterns of the genomic DNA cut with restriction endonucleases and then fractionated on a pulsed-field gel. Unfortunately, the DNA complexity in natural *Epulopiscium* sp. type B populations prevented the resolution of distinct bands from genomic DNA digests. Instead, less direct methods were used to estimate genome size and copy number.

As a proxy for genome copy number, quantitative PCR (qPCR) targeting single-copy house-keeping genes, revealed that *Epulopiscium* sp. type B cells contained an extraordinary number of genome copies (Mendell et al. 2008). Copy number varied from cell to cell, and individuals yielded tens of thousands, to hundreds of thousands of copies of single genes in qPCR assays. Prior to qPCR, cells were photographed so measurements could be used to estimate cell volume. The cells varied in volume by almost 10-fold and gene copy number positively correlated with cell size across this range (Mendell et al. 2008). Quantitative multiplex PCR was not feasible in this study and only one marker could be reliably quantified from a single cell. With extracted genomic DNA from *Epulopiscium* sp. type B populations and the use of highly specific Taqman probes, multiple unlinked single-copy genes were interrogated instead. Although the investigation was limited in scope, all single-copy genes recovered similar results; only a replication origin-linked marker (*dnaA*) yielded significantly higher copy numbers than the other markers tested. The results supported the model that these giant cells contained a typical-sized genome for a bacterium, and moreover *Epulopiscium* sp. type B is highly polyploid throughout its life cycle (Mendell et al. 2008). More recently, chromosomal regions were visualized in intact cells using fluorescent in situ hybridization (FISH) which confirmed that epulos, including *Epulopiscium* sp. type B, are polyploid throughout their life cycle (Hutchison et al. 2018). A high-quality draft genome derived from an *Epulopiscium* sp. type B population has confirmed that these cells possess a typical bacterial genome comprised of a circular chromosome of approximately 3.28 Mbp.

## Unusual DNA Metabolism and Management of Chromosomes in *Epulopiscium* sp. type B

Most bacterial genomes contain one or two covalently closed, circular chromosomes. Although bacterial binary fission is conceptually simple, cells need to carry out chromosome segregation in such a way as to ensure that each daughter cell receives a complete complement of genomic material (Badrinarayanan et al. 2015). Within a bacterial cell, the chromosome(s) form a nucleoprotein complex called the nucleoid. For rapidly growing, rod-shaped bacteria, chromosome orientation in a cell follows the management of the chromosomal origin of replication, *oriC*. Bacterial chromosome replication generally begins at this single site, which is flanked by binding sites for the replication initiation protein DnaA and for proteins involved in partitioning the chromosomes (Ekundayo and Bleichert 2019). Proteins involved in replication, including DnaA, are generally coded for near the replication origin (Ogasawara and Yoshikawa 1992). Once replication begins, partitioning proteins bind origin-proximal sites and guide the replication origins toward opposite ends of the cell (Shen and Landick 2019). Nucleoid associated proteins condense and organize parts of each chromosome and nucleoid occlusion proteins prevent cytoplasmic fission from damaging the chromosomes as they segregate (Badrinarayanan et al. 2015; Dame et al. 2020).

Surprisingly, the physical location of a specific genetic locus within a bacterial cell (i.e., *E. coli, B. subtilis, Caulobacter crescentus* etc.) corresponds with the position of the locus on the chromosome (Viollier et al. 2004; Gitai et al. 2005). Therefore, the orientation of a chromosome in a bacterium is generally predictable. Physical constraints, DNA condensation proteins and entropic forces likely contribute to the ordered positions of chromosomes and their segregation within a bacterium (Jun and Wright 2010). These forces are important for the organization of chromosome copies in polyploid bacteria as well (Jain et al. 2012; Hutchison et al. 2018) but how a cell manages multiple copies of a chromosome is poorly understood. Further, how *Epulopiscium* determines which chromosome copies will be inherited is unknown.

The *Epulopiscium* sp. type B genome contains many of the common partitioning and condensation proteins of bacteria (Hutchison et al. 2018). However, for *Epulopiscium* sp. type B, chromosomes are not subjected to physical confinement, except perhaps in very small offspring cells. The peripheral arrangement of DNA in large cells may be dictated in part by transertion, the linked processes of transcription, translation, and insertion of membrane proteins that can occur in bacteria that tethers a chromosome to the cell membrane (Roggiani and Goulian 2015). Chromosome positional studies in large *Epulopiscium* sp. type B cells using FISH suggested that chromosomes in large epulos are subject to some of the same positional controls as more typical-sized cells (Hutchison et al. 2018). Chromosomal replication origins labeled with FISH were found to be regularly spaced around the cell periphery, but when multiple loci were simultaneously localized, chromosomes in a mother cell appeared to be more relaxed and less positionally constrained than what is observed in bacteria with only a few chromosome copies. The chromosomes inside small offspring were significantly more closely packed than in the mother cell. Based on FISH foci counts, equivalent numbers of chromosomes were partitioned into offspring within a mother cell (Hutchison et al. 2018). Despite the tethered appearance of pole-associated DNA, chromosomes did not appear to take on a particular orientation with respect to the cell poles. Since more than 100 chromosome copies are captured in an offspring cell when the mother cell divides asymmetrically, selection for highly effective chromosome compaction and the need to maintain chromosome order are likely relaxed in *Epulopiscium* sp. type B compared to small bacteria.

**Fig. 4:**
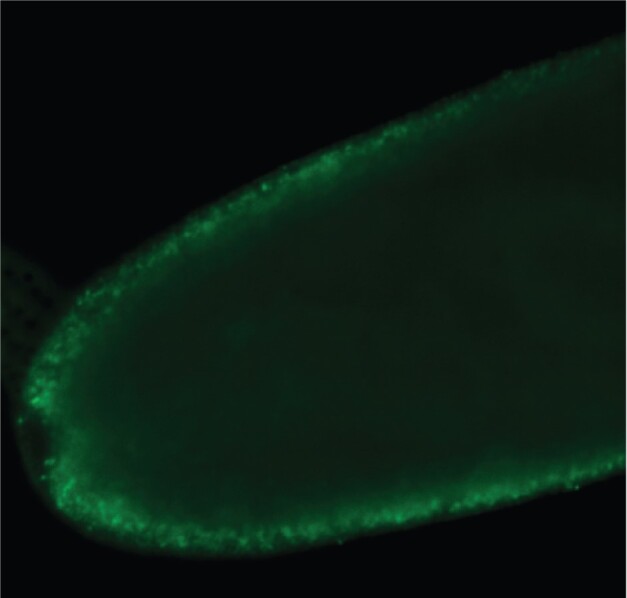
After intracellular offspring are formed, DNA continues to replicate in the terminally differentiated mother cell. To follow the fate of DNA in *Epulopiscium* cells, BrdU was introduced to live cells within the fish intestinal tract. Immunolocalization of the BrdU showed DNA replication had occurred within growing offspring cells but replication was also taking place in mother-cell DNA. Shown here is a medial optical section of the tip of a mother cell. BrdU immunolocalization is shown in green.

As mentioned above, *Epulopiscium* sp. type B mother cells appear to undergo a programmed cell death just prior to offspring emergence (Ward et al. 2009). Mother-cell DNA diminishes near the end of its life and the cell envelop loses its structural integrity. Nucleic acids are rich in carbon, nitrogen, and phosphate. The ability to retain at least some of these organic compounds and provide them to the growing offspring would be advantageous (Ward et al. 2009). Looking for evidence of transfer of DNA, oligonucleotides or even nucleotides between an *Epulopiscium* mother cell and its offspring is technically challenging. One possible approach could employ pulse-labeling of DNA with a nucleotide analog that could be tracked through development. With three generations present in some stages of development (see fig. 3*D*), clear patterns of transfer could be obscured. Nevertheless, pulse-labeling proved to be highly informative in revealing aspects of DNA metabolism in *Epulopiscium* sp. type B cells (Ward et al. 2009). The nucleotide analog bromodeoxyuridine (BrdU) is taken up by most cells, phosphorylated by thymidine kinase, and incorporated into DNA by DNA polymerase. BrdU labeling has been used in eukaryotic cells to detect sites of replication and it has been used in bacterial populations to tag active cells (Lewis and Errington 1997; Urbach et al. 1999; Pernthaler et al. 2002; Ward and Angert 2008). In *Epulopiscium* sp. type B cells, it was effective in labeling locations of newly replicated DNA (Ward et al. 2009). As expected, evidence of replication was seen in nucleoids throughout small offspring cells. Surprisingly, BrdU was also incorporated into chromosomes in mother cells that contained large offspring (fig. 4). This led to the hypothesis that mother-cell nucleoids have an important somatic role and continue to replicate even in terminally differentiated mother cells (Ward et al. 2009). These mother-cell chromosomes likely support the transcription of genes important for mother-cell metabolism and offspring growth, and thus have a somatic role in the lifecycle.

## Limited Inheritance of Genome Copies in *Epulopiscium* sp. type B

Mutations, virus-mediated transduction and horizontal gene transfer may all contribute to the genetic diversity of a bacterial population. With its unusual reproductive strategy, genetic novelty acquired in mother-cell chromosomes in *Epulopiscium* sp. type B would likely be lost, as most of the genome copies are held within a terminally differentiated cell (Arroyo et al. 2019). Genetic changes in chromosomes near the poles of the mother cell would be maintained in the population, if they are packaged into offspring primordia. This daily genetic bottleneck could potentially have a negative impact on *Epulopiscium* sp. type B populations.

The interactions between *Epulopiscium* sp. type B and its host may further impact *Epulopiscium* population diversity. Like many fish species, *N. tonganus* adults do not tend their eggs (Randall 2001; Doherty et al. 2004). When eggs hatch, the larval offspring join the pelagic zooplankton. Within 2–3 months, larval *N. tonganus* reach a few centimeters in length, and settle on a reef. The transition from larval to juvenile fish, and then to adult, involves substantial changes in gut morphology and the diet of these fish. Juvenile fish obtain their gut microbiota from their environment and *Epulopiscium* spp. are most likely acquired by eating feces of adult fish (Clements 1997). Adult and juvenile *N. tonganus* school together so juvenile fish may have regular access to live *Epulopiscium* sp. type B. Unlike spore-forming epulos, *Epulopiscium* sp. type B may only survive for a limited amount of time outside of the host intestinal tract. The immediate need to find a receptive host may present another bottleneck that could impact population diversity and resilience. One additional factor that may influence *Epulopiscium* sp. type B populations is retention of cells by the host fish. It is not known how long an *Epulopiscium* sp. type B lineage (mother, daughter, granddaughter, great-granddaughter…) remains associated with an individual fish or what proportion of the population is expelled on a regular basis. Surgeonfish, including *N. tonganus*, are long lived and may reach ages of 20–45 years (Choat and Axe 1996). Lineages of gut symbionts are potentially retained for decades and the behavior of a host fish would dictate the influx of new individuals and new genes.

*Epulopiscium* sp. type B populations face a number of barriers to maintaining diversity. Surveys of 16S rRNA gene sequences suggested that *Epulopiscium* sp. type B populations harbored little genetic diversity (Angert and Clements 2004; Mendell et al. 2008). A more recent multilocus sequence type (MLST) analysis of cells was used to investigate population diversity using higher resolution data sets (Arroyo et al. 2019). Cells were taken from several individual hosts inhabiting reef systems in and around Lizard Island, Australia. For the MLST study, individual bacteria were hand-selected, cleaned, and subjected to whole genome amplification (WGA). From the WGA DNA, the 16S rRNA genes, along with seven single-copy genes, were amplified by PCR. The 16S rRNA gene sequences were used only for quality control in this study because *Epulopiscium* sp. type B cells are known to harbor multiple rRNA operons (Mendell et al. 2008). The single-copy gene sequences were concatenated and used to explore population diversity (Arroyo et al. 2019). Of the 113 *Epulopiscium* sp. type B cells examined, 88 sequence types were recovered but these symbiont sequence types were not randomly distributed among the 12 fish examined. All but one of the host-associated subsets of *Epulopiscium* sp. type B cells, showed high haplotype diversity. Furthermore, within-host factors influenced symbiont populations. Significant codiversification links were found between individual hosts and their associated symbionts in both Parafit and Principal Coordinates Analysis. However, only a few significant Parafit links in these data contributed to the overall pattern. Together with the observation that some healthy adult *N. tonganus* collected in this study area do not appear to harbor *Epulopiscium* sp. type B cells, these results indicated that the symbionts are dependent on their host, but the host has a facultative relationship with *Epulopiscium* sp. type B (Arroyo et al. 2019).

## Mechanisms to Maintain Genetic Diversity in *Epulopiscium* sp. type B Populations

**Fig. 5:**
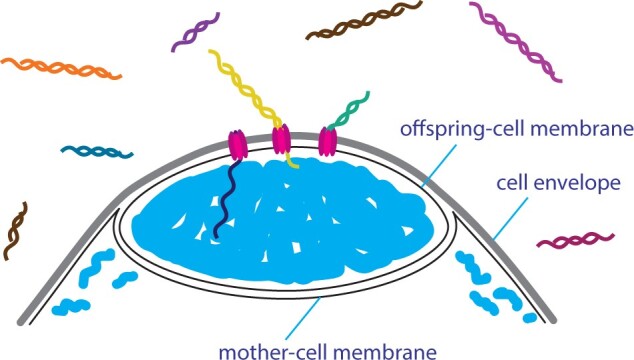
Genetic diversity in *Epulopiscium* sp. type B populations is likely maintained by recombination. A model of inheritance of diverse DNA fragments via congression is shown here. Offspring emerging from their mother cells may be in the process of polar division or harbor pole-associated offspring primordia. Before engulfment is complete, the chromosomes within these primordia would be separated from the environment by only one cell membrane, as illustrated here. If like *B. subtilis*, DNA uptake proteins (competence proteins) assemble at cell poles, newly acquired DNA would be transported into the polar offspring primordia. In this image, fragments of DNA released by the synchronized lysis of diverse mother cells are shown as different colored double helices. Competence proteins are shown as purple transport complexes bringing in single-stranded DNA into the polar offspring. Cell membranes are show as black outlines, the cell envelop is shown in gray, and *Epulopiscium* nucleoids are shown as thick light blue lines.

Based on analyses of the MLSA surveys, recombination is more important than simple mutations for maintaining genomic diversity in *Epulopiscium* sp. type B populations (Arroyo et al. 2019). Unexpectedly, these populations exhibit linkage disequilibrium despite this reliance on recombination. A model to reconcile these two observations is based on features common to many bacterial transformation systems. Competent bacterial cells can take up multiple, unlinked fragments of DNA and incorporate them into their genome at surprisingly high frequencies (Erickson and Copeland 1973; Dalia et al. 2014; Mell et al. 2014). In bacteria, transformation of multiple, unlinked fragments simultaneously is referred to as congression (Erickson and Copeland 1973). Residual mother-cell DNA, released at the time that offspring emerge, would provide diverse, unlinked fragments available for uptake by competent cells (Arroyo et al. 2019). *Epulopiscium* sp. type B contains orthologs for many of the *B. subtilis* competence genes, which produce proteins needed for processing and uptake of environmental DNA and recombination (Dubnau and Blokesch 2019). Specifically, the genome codes for *comC, comEA, comEC, comGB, recA, ssb* and appears to contain more divergent homologs of *comGA* and *comFA*. Although genes required for assembly and anchoring of a DNA uptake pilus are present in *Epulopiscium* sp. type B, a convincing ortholog for the *comGC* pilin gene has not been identified. An alternative DNA uptake pilus may be used by *Epulopiscium* sp. type B, and the genome encodes an ortholog for a type II secretion system pilin, which is used for DNA uptake in other Firmicutes (Muschiol et al. 2015). In *B. subtilis* and some other competent bacteria, the DNA uptake machinery is located at the poles of a competent cell (Chen et al. 2005; Dubnau and Blokesch 2019). If this is also the location of competence machinery in *Epulopiscium* sp. type B cells, DNA would be brought into offspring primordial cells or in the vicinity of pole-associated chromosomes (fig. 5). This location would be advantageous because it would only require the DNA to traverse a single cell membrane. DNA entering *Epulopiscium* sp. type B would likely recombine with germline chromosomes and generate new sequence combinations in developing offspring.

## Conclusions

As with any bacterium, novel functions may be hidden among the genes unique to *Epulopiscium* sp. type B. It is noteworthy that although *Epulopiscium* sp. type B is an exceptional bacterium, common bacterial mechanisms of cell cycle control and host/symbiont interactions appear to be operating and are flexible enough to support this unusual bacterium. Selection to maintain a productive association with a host has been a major force in shaping the developmental and cellular biology of *Epulopiscium* sp. type B. Being a giant and highly motile bacterium, may allow *Epulopiscium* sp. type B to maintain its place in the intestinal tract and a long-term relationship with an individual fish (Arroyo et al. 2019). In this competitive environment, being large may confer several additional benefits. The intestinal ecosystem within *N. tonganus* supports a diverse microbial community, including an abundance of flagellates and ciliates. Giant *Epulopiscium* sp. type B cells may avoid some predation by ciliates like *Balantidium jocularum*, but not entirely (Grim 2006). Furthermore, sequestration of nutrients (e.g. oligosaccharides, etc.) within the cytoplasm of these giants may provide a competitive advantage as it appears to in *E. coli* (Gallet et al. 2017). However, the unusual cellular biology of *Epulopiscium* sp. type B may have driven the emergence of a novel form of reproduction that limits genetic inheritance.

Multiple essential cellular mechanisms and external factors collaborate to provide a means of overcoming genetic bottlenecks that have the potential to endanger the diversity of *Epulopiscium* sp. type B populations. The host provides a carbon-rich environment and dependable source of energy, needed to maintain polyploidy throughout the lifecycle of *Epulopiscium* sp. type B. In addition, densely populated regions of the host intestinal tract ecosystem allow for close associations, which may help facilitate gene exchange. The synchronized development of offspring appears to be important for effective transfer of residual chromosomal DNA from dying mother cells to offspring primordia. Competence machinery at the poles of *Epulopiscium*, as seen in other bacteria, would enable transfer specifically to offspring genomes. If the competence machinery were randomly distributed around the cell periphery, it would be less likely that transforming DNA would be incorporated into the germline (offspring) chromosomes. Here “simple” modifications of a developmental program, and a lifestyle that affords predictable and reliable access to usable carbon and energy, can lead to the evolution of giant microorganisms with complex germline dynamics.
